# An investigation of factors affecting compassion levels of midwives

**DOI:** 10.18332/ejm/146586

**Published:** 2022-05-18

**Authors:** Tuğba Yilmaz-Esencan, Ayça Demir-Yildirim, Sena-Nur Uzun

**Affiliations:** 1 Department of Midwifery, Faculty of Health Sciences, Üsküdar University, Istanbul, Turkey; 2 Medipol University Hospital, Istanbul, Turkey

**Keywords:** midwife, midwifery care, ethics, compassion, holistic care

## Abstract

**INTRODUCTION:**

The profession of midwifery is seen as a genuinely individual-centric catalyst transforming compassion to care. Due to this, compassionate care should be the basis of midwives’ care, especially for those who care for women and children. This research investigated the compassion levels of midwives to determine the factors affecting them.

**METHODS:**

This descriptive research was conducted between 10 February and 29 May 2020, with 119 midwives working in a training and research hospital in Istanbul. The data were collected using the ‘Compassion Scale’ and analyzed using the Mann-Whitney U test, Kruskal-Wallis test and logistic regression model.

**RESULTS:**

The total mean score on the compassion scale of midwives participating in the study was found to be 71.46 ± 9.60. Sociodemographic data and vocational belongings of participants were compared with the mean scores of the compassion scale sub-dimensions. It was determined that as the age of the midwives increased, they showed more kindness and awareness of compassion (p<0.021, p<0.023, respectively). It has been determined that as the education level of midwives decreases, their compassion level also decreases and they become more insensitive (p<0.044). It was found that being married increased the kindness (p<0.023) and common humanity characteristics (p<0.032) that affect compassion. It was found that the levels of kindness (p<0.020) and awareness (p<0.048) increased for those who had children, and compassion was associated with having a child. In addition, a statistically significant relationship was found between the professional commitment and kindness of the midwives participating in the study (p<0.034). It was found that midwives’ knowledge of rules related to professional ethics increased their compassion scale scores by 1.2.

**CONCLUSIONS:**

Midwifery is a profession with a high sense of spirituality. However, professional belonging, love of the profession and professional development positively affect midwives’ levels of compassion. Improving midwives’ feelings of belonging and their professional development will also contribute to the quality of maternal and infant health.

## INTRODUCTION

Compassion has existed and developed since the beginning of humanity, and it is one of the human emotions^1^. The concepts of ‘care’, ‘sympathy’, ‘empathy’ and ‘compassion’ co-exist from time to time in literature. At the basis of these concepts, which are all separate, is the sense of compassion^2-4^.

For health professionals, compassion is an emotion that prevents patients to be affected by negative experiences by exhibiting a supportive attitude and helping them. Every individual in any part of the world needs a sense of compassion; however, patients need compassion more than anyone else^5,6^. The care that patients need is not only for their illness, but also includes an emotional well-being process as a reflection of the situation. For this reason, despite technically perfect care may be given, if emotions are missed in the treatment, the desired results cannot be achieved and the treatment process remains incomplete in this respect^5,7^. It is observed in the literature that individuals prefer compassionate healthcare^7,8^. A study with 53 patients at a city hospital in Canada found sympathy essentially useless while finding empathy and compassion positive and beneficial, with compassion being the most preferred concept^9^. In another study involving 100 patients hospitalized in intensive care units and 79 nurses working in these units, compassion was found to be essential in the care. During care services, one of the primary needs of the patients is a compassionate approach. It is noted that without compassionate care, individuals are merely passive recipients of given care rather than actively participating in their own care^10^.

Healthcare parameters should evaluate the individuals holistically and respond to all expectations and needs, including full emotional support. This emotional connection is the most critical feature that distinguishes healthcare services from other kinds of services^5,7,11^. The literature also emphasized that compassionate care practices positively influence symptom management in operating rooms, surgical clinics, intensive care units, maternity units, and other long-term care settings^7,8,12^. Midwifery care includes especially emotional attachment and compassion. Compassionate midwifery care requires a holistic approach and constitutes an essential part of the treatment^12-14^.

Compassion, being one of the critical criteria that increase satisfaction in midwifery care, ensures that midwives establish therapeutic relationships and increase the effectiveness of care by improving its quality. The profession of midwifery is seen as a genuinely individual-centric catalyst, transforming compassion to care^5,12,15^. It is inherent in the profession of midwifery that midwives, who provide community-based services and work with women, have advanced feelings of compassion. However, there are no relevant evidence-based studies in the literature. Midwives and other health workers are at the hospital every day and they are familiar with the environment; however, being in the hospital environment is unusual for most people. It constitutes a startling and stressful process for them. The nature of birth may be negatively affected by this process. In addition, if an individual has previously gone through negative experiences, they may be afraid of experiencing the same things again. For instance, having a negative birth experience can affect the next birth as well. Through compassionate midwifery care given at this stage, women feel less anxiety and more confidence^8,15-17^. These conclusions also reveal the concept of compassion and humane birth in the clinic, but need to be proven with further research.

The relationship between compassion and midwife clinicians is described as providing individual-specific care and counselling by combining medical care and scientific knowledge on human foundations^18-20^. It is also noted that care blended with empathy and compassion in midwifery positively affects the path of pregnancy and childbirth^21,22^. The journey of a woman who gives birth contains empathy and compassion in its nature. Because this journey goes through stages of significant emotional changes, as a requirement of the physiological process, it also involves the midwife, who has a central role in the journey^23^. Moreover, according to many international organizations related to midwifery, compassion is at the center of midwifery care^24-26^.

Because compassion has such an importance for the profession of midwifery, our research aims to examine the factors affecting the compassion levels of midwives. The research seeks answers to the following questions: 1) ‘Do sociodemographic data of midwives influence their compassion levels?’, and 2) ‘Is there a relationship between the midwives’ knowledge of professional ethics codes and their compassion levels?’.

## METHODS

### Study design and population

The study was conducted as descriptive cross-sectional research. The study was conducted between February and May 2020 in a training and research hospital located on the Anatolian side of Istanbul. The study population comprised all midwives who worked in this hospital and met the criteria for admission to the research. The number of midwives who were active employees at the hospital where the research was conducted was determined to be 120. A power analysis was performed to ensure representation of the universe of study. Power analysis was carried out for the sample, and 118 people were found. The analysis was conducted using G*Power 3.1. The research was carried out with 119 people due to one person failing to complete the scale.

Criteria for inclusion of volunteers in the research were determined as follows: being an employee at the hospital where the research is being conducted, being a midwife, being an active employee, volunteering to participate in the research, and completing the surveys in their entirety.

### Data collection tools and collection of data

Research data were collected with the Information Form developed by the researchers through literature^4-6,12^ review and via face-to-face interview method using the Compassion Scale. At the data collection stage, completion of the forms was taken as a criterion, and one incomplete form was removed from the data set.

The introductory Information Form consists of a total of 22 questions. The distribution of questions was as follows: four on sociodemographic information, seven on vocational knowledge, two on professional and ethical codes, and nine on compassion.

The Compassion Scale was developed by Pommier^27^ and adapted to Turkish by Akdeniz and Deniz^4^. It is a 5-point Likert-type scale that contains 24 items and can measure compassion for others in six dimensions: [kindness (6, 8, 16, 24), indifference (2, 12, 14, 18), common humanity (11, 15, 17, 20), separation (3, 5, 10, 22), mindfulness (4, 9, 13, 21), and disengagement (1, 7, 19, 23)]. Scoring on the scale is as follows: 1=never, 2=rarely, 3=occasionally, 4=often, and 5=always. The scores on the scale’s indifference, separation and disengagement sub-dimensions are inverted before calculation. The lowest score on the scale is 24, while the highest score is 120. As the score increases, the level of compassion increases. As a result of a Confirmatory Factor Analysis (CFA) of the scale conducted by Akdeniz and Deniz^4^, the existence of six dimensions that constitute the structure of the scale was confirmed. The Cronbach alpha internal consistency coefficient for the scale was found to be 0.85. In the validity and reliability study of the scale conducted in Turkish by Akdeniz and Deniz^4^, the correlation coefficient between the scores of 41 people who filled in the form in English and filled in the form again in Turkish 25 days later was found to be r=0.78 (p<0.01) and the scale was thus determined to be suitable for Turkish culture^4^.

### Evaluation of data

SPSS 23 Pack software was used for data analysis. Statistical calculations were performed using ANOVA, t-test and chi-squared when variables were distributed normally; otherwise, Mann-Whitney U, Kruskal-Wallis and logistic regression analysis tests were used. Statistical significance was set at p<0.05.

## RESULTS

A total of 119 midwives participated in the study. Sociodemographic data of participants are as follows: more than a half (52.1%) of the participants were aged 26–30 years. The majority of the midwives involved in the study (60.5%) had 0–5 years of work experience, while 10.1% had >26 years. The research site is the first place of employment for midwives with fewer years of work experience (72.3%). When looking at the reasons participating midwives gave for choosing the profession, 27.7% cited a liking of helping others, 25.2% cited the likelihood of finding employment, and 10.1% cited their centralized exam scores for their preference (Table 1).

**Table 1:** Sociodemographic data of participants, Istanbul in Turkey, 2020 (N=119)

*Characteristics*	*Categories*	*n (%)*
**Age** (years)	19-25	17 (14.3)
	26-30	62 (52.1)
	31-35	15 (12.6)
	36-40	5 (4.2)
	41-45	13 (10.9)
	≥46	7 (5.9)
**Education level**	High school	8 (6.7)
	Associate degree	7 (5.9)
	Bachelor’s degree	91 (76.5)
	Graduate degree	13 (10.9)
**Marital status**	Married	50 (42.0)
	Single	69 (58.0)
**Has children**	Yes	32 (26.9)
	No	87 (73.1)
**Reason for choosing the profession**	I do not know	19 (16.0)
	Likelihood of finding employment	30 (25.2)
	Request of family	14 (11.8)
	A liking of helping others	33 (27.7)
	Exam score	12 (10.1)
	Other	11 (9.2)
**Years of work experience**	0-5	72 (60.5)
	6-10	15 (12.6)
	11-15	9 (7.6)
	16-20	4 (3.4)
	21-25	7 (5.9)
	≥26	12 (10.1)
**Choosing the profession of midwifery again**	Yes	74 (62.2)
	No	45 (37.8)

When the participants’ thoughts on code of ethics for midwives and care practices were examined, the majority of the participating midwives (95%) stated that they knew about professional ethics codes. Among the most well-known ethical codes are the principles of doing good (89%), equity (87%), respect for human dignity (95%) and truthfulness (85%), while altruism (27%), autonomy (28%) and esthetics (39%) are among the least popular. Midwives participating in the research stated that during treatment, they would give care in a comforting way and provide support so that pregnant women could cope with their fears (97.5%) (Table 2).

**Table 2:** Participants’ thoughts on code of ethics for midwives and care practices, Istanbul in Turkey, 2020 (N=119)

*Thoughts*		*n (%)*
**Knowledge of ethical codes**	Yes	113 (95.0)
	No	6 (5.0)
**Providing care in accordance with ethical codes**		
Doing good	Yes	89 (74.8)
	No	30 (25.2)
Altruism	Yes	27 (22.7)
	No	92 (77.3)
Equity	Yes	87 (73.1)
	No	32 (26.9)
Respect for human dignity	Yes	95 (79.8)
	No	24 (20.2)
Justice	Yes	81 (68.1)
	No	38 (31.9)
Truthfulness	Yes	85 (71.4)
	No	34 (28.6)
Holism	Yes	58 (48.7)
	No	61 (51.3)
Autonomy	Yes	28 (23.5)
	No	91 (76.5)
Esthetics	Yes	39 (32.8)
	No	80 (67.2)
**Feelings experienced while giving care**		
When a pregnant woman is scared	I support and comfort her	116 (97.5)
	I do not pay attention at all	3 (2.5)
When a pregnant woman talks about her problem	I care and listen patiently	108 (90.8)
	I do not listen because I do not think it concerns me	11 (9.2)
	I leave her alone	19 (16.0)
	I am supportive	100 (84.0)

The total mean score on the compassion scale was 71.46 ± 9.60 (range: 53–120). There are six dimensions of the scale, collected under the headings of kindness, indifference, common humanity, separation, mindfulness, and disengagement. Sociodemographic data and vocational belonging of participants were compared with the mean scores of the compassion scale sub-dimensions. There is a significant association between the age of participants and the sub-dimensions of ‘kindness’ and ‘mindfulness’ (χ^2^=13.315, p<0.021 and χ^2^=12.991, p<0.023, respectively). Midwives have more caring and conscious awareness as their age increases. As midwives’ level of education decreases, sub-dimension scores of ‘indifference’ and ‘separation’ increase at a statistically significant level (χ^2^=48.091, p<0.044 and χ^2^=11.622, p<0.009, respectively). Another statistically significant association was found between the ‘kindness’ sub-dimension scores of the midwives participating in the study, and choosing the same profession again (χ^2^=4.501, p<0.032) (Table 3).

**Table 3:** Comparison of sociodemographic data of participants to the sub-dimensions of the scale, Istanbul in Turkey, 2020 (N=119)

*Characteristics*	*n*	*Kindness*	*Indifference*	*Common humanity*	*Separation*	*Mindfulness*	*Disengagement*
		*Mean rank*	*Mean rank*	*Mean rank*	*Mean rank*	*Mean rank*	*Mean rank*
**Age** (years)							
19–25	17	40.97	74.03	52.82	71.53	46.88	74.85
26–30	62	58.14	61.91	58.40	59.95	55.55	58.49
31–35	15	68.57	49.80	69.60	55.07	81.00	50.67
36–40	5	92.60	49.60	69.80	54.90	86.60	57.70
41–45	13	60.58	49.04	64.35	49.81	67.08	57.27
≥46	7	80.00	58.64	55.93	65.57	54.14	64.0
Statistical analysis*							
χ^2^		13.315	6.204	2.779	3.689	12.991	4.69
p		0.021	0.287	0.734	0.595	0.023	0.454
**Education level**							
High school	8	60.64	73.44	52.69	62.94	49.19	63.31
Associate degree	7	63.79	83.71	77.29	85.36	56.36	81.36
Bachelor’s degree	91	59.89	59.52	60.10	61.52	60.70	59.20
Graduate degree	13	58.35	42.35	54.46	33.92	63.69	52.08
Statistical analysis*							
χ^2^		0.120	48.091	2.490	11.622	1.069	3.589
p		0.989	0.044	0.477	0.009	0.785	0.309
**Marital status**							
Married	50	68.39	59.76	67.92	53.50	63.51	57.27
Single	69	53.92	60.17	54.26	64.71	57.46	61.98
Statistical analysis*							
χ^2^		**5.184**	0.004	**4.613**	3.109	0.908	0.555
p		**0.023**	0.948	**0.032**	0.078	0.341	0.456
**Has children**							
Yes	32	72.00	54.91	68.41	54.28	70.22	54.14
No	87	55.59	61.87	56.91	62.10	56.24	62.16
Statistical analysis*							
χ^2^		**5.383**	0.972	2.638	1.222	**3.907**	1.298
p		**0.020**	0.324	0.104	0.269	**0.048**	0.255
**Reason for choosing the profession**							
I do not know	19	60.61	57.66	60.05	63.39	60.21	55.53
Likelihood of finding employment	30	55.02	68.23	69.98	67.82	60.25	63.53
Request of family	14	58.29	66.18	54.39	51.82	61.21	62.82
A liking of helping others	33	64.65	54.45	55.12	54.65	58.48	55.02
Exam score	12	54.21	55.42	68.21	63.92	64.83	68.04
Other	11	67.09	55.36	45.50	55.00	56.68	60.68
Statistical analysis*							
χ^2^		2.103	3.524	6.258	3.747	0.428	2.131
p		0.835	0.612	0.282	0.586	0.995	0.831
**Years of work experience**							
0–5	72	57.01	61.31	60.67	60.30	55.35	59.58
6–10	15	56.23	59.27	49.70	62.03	63.30	53.03
11–15	9	59.67	65.50	69.06	58.72	72.22	70.28
16–20	4	66.88	59.00	81.63	73.75	76.13	73.88
21–25	7	77.29	40.36	47.93	42.00	85.00	51.71
≥26	12	70.50	60.71	61.92	62.54	54.63	63.75
Statistical analysis*							
χ^2^		3.809	2.666	4.517	2.718	7.543	2.687
p		0.577	0.751	0.478	0.743	0.183	0.745
**Choosing the profession of midwifery again**							
Yes	74	65.19	58.20	60.53	60.01	64.14	59.07
No	45	51.47	62.96	59.13	59.98	53.20	61.53
Statistical analysis*							
χ^2^		**4.501**	0.541	0.046	0.000	2.860	0.141
p		**0.034**	0.462	0.830	0.996	0.091	0.701

* Kruskal-Wallis test, p<0.005.

The relationship of midwives’ vocational belonging and thoughts on the principles of professional ethics codes to the total mean score on the compassion scale was examined. As for the association between mean compassion scale scores and professional ethics codes, ‘justice and truthfulness’ were the only principles of professional ethics to have a statistically significant relationship to providing care (p=0.050, p=0.006, respectively). No statistically significant association was found between midwives’ sociodemographic data and their mean scores on the compassion scale (Table 4).

**Table 4:** Difference between the sociodemographic data of participants and mean compassion scale scores, Istanbul in Turkey, 2020 (N=119)

*Characteristics*	*Categories*	*n*	*Scale score mean± SD*	*p*
**Age** (years)	19-25	17	71.94 ± 7.05	0.926
	26-30	62	70.96 ± 9.32	
	31-35	15	72.86 ± 14.87	
	36-40	5	74.60 ± 9.28	
	41-45	13	70.15 ± 6.65	
	≥46	7	72.42 ± 10.65	
**Education level**	High School	8	70.37 ± 9.24	0.117
	Associate degree	7	78.57 ± 7.18	
	Bachelor’s degree	91	71.53 ± 9.88	
	Graduate degree	13	67.76 ± 7.40	
**Has children**	Yes	32	71.65 ± 8.38	0.894
	No	87	71.39 ± 10.06	
**Reason for choosing the profession**	I do not know	19	71.84 ± 14.03	0.576
	Likelihood of finding employment	30	73.66 ± 6.50	
	Request of family	14	70.92 ± 7.82	
	A liking of helping others	33	69.96 ± 11.09	
	Exam score	12	72.91 ± 8.46	
	Other	11	68.36 ± 9.60	
**Years of work experience**	0-5	72	71.85 ± 8.51	0.559
	6-10	15	69.80 ± 10.47	
	11-15	9	76.22 ± 17.79	
	16-20	4	76.00 ± 7.61	
	21-25	7	69.57 ± 8.05	
	≥26	12	72.00 ± 8.20	
**Care with professional ethics codes**	Altruism	27	69.48 ± 7.38	0.225
	Doing good	89	71.11 ± 8.78	0.496
	Equity	87	70.56 ± 7.77	0.093
	Respect for human dignity	95	71.09 ± 8.91	0.409
	Justice	81	70.28 ± 7.55	**0.050**
	Truthfulness	85	69.95 ± 7.24	**0.006**
	Holism	58	70.10 ± 7.56	0.113
	Autonomy	28	72.28 ± 6.75	0.606
	Esthetics	39	72.84 ± 6.96	0.224

Logistic regression analysis was performed to look at the strength of the relationship between the mean scores of the compassion scale and the sociodemographic factors of the midwives. In the regression analysis, the odds ratio of the variable was found to be 0.862. The average score of those who did not know the ethics codes of midwifery from the compassion scale is 1.2 (1/0.862) times less than those who did (Table 5).

**Table 5:** Influence of participants’ sociodemographic data on the compassion scale score, Istanbul in Turkey, 2020 (N=119)

*Variable*	*OR (Exp b)*	*95% CI for Exp(b)*		p^*^
		*Lower*	*Upper*	
Age	1.005	0.967	1.045	0.786
Education level	0.972	0.927	1.020	0.248
Marital status	1.010	0.972	1.049	0.617
Choosing the profession again	3.027	0.983	1.073	0.231
Membership to a professional association	1.012	0.973	1.053	0.557
Knowledge of professional ethics codes	**0.862**	0.790	0.941	**0.000**

* p<0.05.

## DISCUSSION

The average score on the compassion scale is 60 out of the highest possible score. In our research, the total mean score on the compassion scale was found to be 71.46 ± 9.60 (range: 53–120). The lowest score one can receive on the compassion scale is 24, while the highest score is 120, and according to the data obtained in our research, midwives’ compassion levels were above average. In a different study conducted in Turkey with 78 midwives working only in the delivery room, the total mean compassion score was 4.19 ± 0.39, and thus relatively high^7^. In our study, the average compassion scale was higher. Literature states that the care provided by midwives with high levels of compassion would significantly contribute to the health of the mother and the child and that compassionate midwifery care would improve preventive health services^7,28-30^. Midwives witness the uniqueness of childbirth and are therefore expected to be more sensitive in terms of compassionate care^7^. Compassion is also central to supportive and holistic midwifery care, which is among professional roles of midwives^29^. Studies emphasize that compassion is one of the most important values that professionals involved in the provision of health services should have^30,31^. Although studies on midwives are limited, other studies with nurses also show high levels of compassion^31-35^. Research findings support the literature data on the level of compassion.

Our research looked at whether sociodemographic data and vocational belonging made a difference in the total mean score on the compassion scale and found a statistical difference between the compassion scale score and level of education (χ^2^=9.721, p=0.021). It is noted that compassion should be encouraged during undergraduate training, especially to healthcare providers whose work centers around care^34^. Of the midwives who participated in our research, 76.5% had a Bachelor’s degree. It was determined that a high level of education contributes to having high levels of compassion. Another study found that compassion-oriented instruction given to medical school students improved their perceptions of compassionate care^32^. In this respect, research data support the findings of the similar studies. As a consequence, the importance of forming the components of midwifery education by taking compassion-oriented care into account, becomes apparent.

Our research did not find a statistical difference between compassion scale scores and age, marital status, or having children (p>0.05). Research conducted by Cingöl et al.^31^ and also a study conducted by İşgör^36^ on university students show similar results with our study. This was thought to be due to the average age of participants of all three studies being close. However, another study conducted with nurses, unlike our research, found a statistically very significant difference between age and compassion scale score means^37^.

Scale sub-dimension means were found to be 17.0 ± 2 for kindness, 7.0 ± 3.7 for indifference, 16.0 ± 3.2 for common humanity, 7.0 ± 3.3 for separation, 17.0 ± 2.9 for mindfulness, and 6.0 ± 3.4 for disengagement. Midwives who participated in our research scored highest on the sub-dimensions of kindness and mindfulness. These data show similarities with other research in the literature^31,38,39^. Judging from the scale sub-dimension scores of the midwives who participated in our research, we can conclude that scoring high in the dimension of kindness is relevant to providing support to people they consider to be in a problematic situation in distress. The high mean score on the mindfulness dimension is thought to be linked to higher listening and communicative skills and proficiency in noticing verbal or non-verbal cues. These data are consistent with the professional significance of communication skills and that providing supportive care has core importance for midwifery. It is known that care provided within the framework of the fundamental values of compassion and respect for the individual improves communication, patient satisfaction, and quality of care^40^. Our research findings support the literature data.

In a study conducted on 227 nurses, compassion scale sub-dimension means were 16.44 ± 2.47 for kindness, 7.44 ± 2.40 for indifference, 15.46 ± 2.80 for common humanity, 7.76 ± 2.29 for separation, 16.03 ± 2.47 for mindfulness, and 7.70 ± 2.23 for disengagement, respectively^41^. These means show similarity with the data of our research. Another study found scale sub-dimension means of midwifery students to be 17.26 ± 2.98 for kindness, 6.90 ± 2.59 for indifference, 16.37 ± 3.05 for common humanity, 16.63 ± 2.94 for mindfulness, and 6.79 ± 2.63 for disengagement^39^. These results are also close to our data. Likewise, our participants scored lowest on the sub-dimension of disengagement, linked to being apathetic in a negative situation. One of the essential elements of midwifery is trust in communication with pregnant women, reflected in our research data. Our research findings also support the literature data in this sense as well.

Our research found a significant relationship between the sub-dimensions of ‘kindness’ and ‘cognitive awareness’ and the age of participants. In contrast, another study found a statistically significant association only between cognitive awareness and age^41^. Another study conducted on 346 health workers found that as the age of nurses increased, the scores on the sub-division of kindness increased^6^. These data are similar to our research data. Nevertheless, another study conducted on 100 male surgeons found a significant relationship between age and service, indifference, common humanity, separation, and cognitive awareness^13^. In a different study, it was noted that generation had no impact on compassion whatsoever^31^. There are also studies on the investigated relationship that have very different outcomes from which we found in our research.

When looking at the impact of education level on the compassion scale sub-dimensions, as the level of education decreases, the scores on the sub-dimensions of ‘indifference’ and ‘separation’ increase in a statistically significant manner. This was associated with the midwifery students’ being taught skills that feed a sense of compassion during the training process. A study conducted with nurses found a relationship between education level and the sub-dimension of mindfulness, while another study found that empathy was associated with age and professional satisfaction^41,42^. A study with midwives found a significant association between age, educational decency, working conditions, and compassion scale score. These data support our research data. A study conducted on 181 midwives found that long working hours and exposure to traumatic birth events negatively affected midwives’ levels of compassion^43^. Unlike our research, it is also seen that there is a relationship between compassion and working conditions and working hours.

In our research, a significant relationship was found between the likelihood of choosing the same profession again and the scale sub-dimension score of ‘kindness’, and this was thought to be related to professional satisfaction. When comparing marital status to scale sub-dimensions, it can be observed that there is a significant relationship between being married and the sub-dimensions of ‘kindness’ and ‘common humanity’. In a different study, it was also noted that the mean separation sub-dimension score was higher in unmarried participants^39^. What differentiates this research from most others in the literature is that it focuses on the relationship between the factors affecting the emotion of compassion and notes that midwives’ knowledge of professional ethics codes increases their sense of empathy by 78%. Ethical principles and professional values in midwifery are known to guide midwives into compassionate care^7,8^. Our research also shows that midwives’ adherence to professional ethics codes is associated with compassionate care.

### Limitations

This study is the first to examine midwives’ levels of compassion in the province of Istanbul. However, it does not represent all midwives in Turkey, as it is limited to midwives working in a training and research hospital located on the Anatolian side of Istanbul. The sample count of the study is small. There could be other variables affecting compassion that have not been collected or analyzed. Therefore, meaningful conclusions are only suggestive.

## CONCLUSIONS

It was found that midwives participating in our research had higher than average levels of compassion. When examining the sub-dimensions, it was observed that they had higher levels of kindness, which reflects a high level of support, and higher levels of mindfulness, which reflects strong communication skills.

Compassion is one of the most important emotions that form the basis of midwifery care. Therefore, having chosen the midwifery profession suggests a high level of compassion in an already compassionate individual. It is expected from the nature of the profession that all midwives provide kind and compassionate midwifery care. Knowledge of the profession’s ethical codes also influences the likelihood of providing kind and compassionate midwifery care by 78%. As a result of our study, it became clear that more research that examines midwives’ levels of compassion is necessary, as is working on different sample groups. It is advised that the results of such research be reflected in vocational training: midwives should be given in-service training on compassion, and this training should also be included in the midwifery curriculum.

## Data Availability

The data supporting this research are available from the authors on reasonable request.
